# Predictors of outcome in myxoedema coma: a study from a tertiary care centre

**DOI:** 10.1186/cc6211

**Published:** 2008-01-03

**Authors:** Pinaki Dutta, Anil Bhansali, Shriq Rashid Masoodi, Sanjay Bhadada, Navneet Sharma, Rajesh Rajput

**Affiliations:** 1 Department of Endocrinology, Postgraduate Institute of Medical Education and Research, Chandigarh-160012, India; 2 Department of Critical Care Medicine, Postgraduate Institute of Medical Education and Research, Chandigarh-160012, India

## Abstract

**Background:**

With the easy availability of thyroid hormone assays, thyroid disorders are now recognised even in a subclinical state. However, patients are still seen with advanced manifestations of the disease, particularly in developing countries. This observational study analysed the predictors of outcome in patients with myxoedema coma and tested the validity of different modules to define morbidity and mortality in these patients.

**Methods:**

Twenty-three consecutive patients with myxoedema coma who presented from January 1999 to August 2006 were studied. The thyroid function test and random serum cortisol were measured in all patients at the time of admission. Patients were given oral or intravenous (IV) thyroxine with intention to treat with the latter according to availability. Various modules that predict outcome, including Glasgow Coma Scale (GCS), Acute Physiology and Chronic Health Evaluation II (APACHE II) score, and Sequential Organ Failure Assessment (SOFA) score, were analysed. SOFA score was repeated every 2 days until the time of discharge or demise.

**Results:**

Twenty-three patients (20 women; 87%) of 59.5 ± 14.4 years of age (range, 30 to 89 years) were seen during the study period. Nine (39%) patients were diagnosed with hypothyroidism for the first time at the time of presentation of myxoedema coma, whereas 14 (70%) were diagnosed with hypothyroidism previously. However, the treatment defaulters presented early to the hospital and had more severe manifestations than *de novo *subjects. Nineteen (82%) had thyroprivic (primary) and 4 (17%) had trophoprivic (secondary) hypothyroidism. Fifteen (65%) patients presented in the winter and in 17 (74%) sepsis was the major accompanying comorbidity. Twelve (52%) had a history of diuretic use, thereby delaying the initial diagnosis. Patients who received oral L-thyroxine had no difference in outcome from those receiving IV thyroxine. Twelve (52%) subjects died and sepsis was the predominant cause of death. Various predictors of mortality included hypotension (*p *= 0.01) and bradycardia (*p *= 0.03) at presentation, need for mechanical ventilation (*p *= 0.00), hypothermia unresponsive to treatment (*p *= 0.01), sepsis (*p *= 0.01), intake of sedative drugs (*p *= 0.02), lower GCS (*p *= 0.03), high APACHE II score (*p *= 0.04), and high SOFA score (*p *= 0.00). However, SOFA score was more effective than other predictive models as baseline and day 3 SOFA scores of more than 6 were highly predictive of poor outcome.

**Conclusion:**

L-Thyroxine treatment defaulters had more severe manifestations compared with *de novo *subjects. Outcome was not influenced by either aetiology or route of administration of L-thyroxine, and SOFA score was the best outcome predictor model.

## Introduction

Myxoedema coma is an uncommon and life-threatening form of long-standing, neglected, untreated hypothyroidism with physiological decompensation [1]. This endocrine crisis usually occurs in elderly women and is precipitated by a secondary insult such as cold exposure, infection, drugs such as sedative-hypnotics, lithium overdoses, and associated systemic diseases [1,2]. Clinically, it is characterised by lethargy, myxoedematous manifestation, and altered sensorium in the form of stupor, delirium, or coma. Due to widespread use of screening tests for thyroid dysfunctions and hence the early diagnosis even at the subclinical state, this entity has become rare in Western countries. In developing countries, recognition of this entity is hampered by its slow onset, lack of knowledge among physicians and patients, and absence of cost-effective guidelines to screen for subclinical thyroid diseases. Therefore, patients presenting with florid manifestations of the disease are not uncommon. The incidence of myxoedema coma in European countries is 0.22 per million per year [3]; however, no such epidemiological data are available from the Indian subcontinent. Review of the literature suggests that most of the cases of myxoedema coma are isolated case reports or a handful of series comprising only a few patients [4-16]. Myxoedema coma, if not appropriately treated, has been associated with progressive multi-organ dysfunction and mortality. The predictors of mortality modules used are Glasgow Coma Scale (GCS) and Acute Physiology and Chronic Health Evaluation II (APACHE II) and Sequential Organ Failure Assessment (SOFA) scores. However, GCS and APACHE II score are assessed only at baseline and do not account for subsequent morbidity. On the contrary, SOFA score assesses the cumulative morbidity and mortality during hospital stay. The present study analysed the presentation and factors predicting outcome of the patients with myxoedema coma and tested the validity of various available outcome predictor models to define morbidity and mortality in these patients.

## Materials and methods

This prospective observational study includes consecutive patients with myxoedema coma who presented to our institute from January 1999 to August 2006. The study was approved by institute's ethics committee, and written informed consent was obtained from relatives of patients. Diagnosis of myxoedema coma was based on altered sensorium ranging from obtundation and stupor to coma, hypothermia (core body temperature of less than or equal to 35°C), a precipitating illness, and low serum level of total or free thyroxine (T_4_) (total T_4 _of less than 58 nmol/L and free T_4 _of less than 10.3 pmol/L, lower limit of reference range). Sick euthyroid syndrome was excluded by careful clinical examinations and appropriate investigations. In all patients with primary hypothyroidism, a thyroid-stimulating hormone (TSH) level of greater than 20 mU/L was taken along with low total and/or free T_4_, and in patients with secondary hypothyroidism, structural abnormalities of hypothalamo-pituitary area along with low total and/or free T_4 _were considered diagnostic. Stupor was defined as a lesser degree of unarousability in which the patient can be awakened by external stimuli, accompanied by motor behaviour leading to the avoidance of uncomfortable or aggravating stimuli. Obtundation was defined as easy arousal and the persistence of alertness for brief periods. Both are always accompanied by some degree of confusion. Altered sensorium was graded by GCS. Hypoventilation was defined as PaCO_2 _(partial pressure of carbon dioxide, arterial) of greater than or equal to 43 mm Hg. Glucocorticoid sufficiency was defined by random plasma cortisol of greater than or equal to 350 nmol/L [9].

A blood sample for thyroid function, random cortisol, hemogram, biochemistry, and arterial blood gas analysis was taken in all patients prior to any therapy. The normal values of different hormones were total triiodothyronine (T_3_) of 0.92 to 2.78 nmol/L, free T_3 _of 0.22 to 6.78 pmol/L, total T_4 _of 58 to 140 nmol/L, free T_4 _of 10.3 to 35 pmol/L, TSH of 0.15 to 4.55 mU/L, and random plasma cortisol of 360 to 900.0 nmol/L. The patients were treated with two regimens of L-T_4_: either an initial loading dose of 500 μg of oral L-T_4 _tablets (Eltroxin; GlaxoSmithKline India, Mumbai through a nasogastric tube followed by 150 μg of maintenance dose or an initial intravenous (IV) loading dose of 200 μg of L-T_4 _sodium Ben Venue laboratories, Inc., Bedford OH 44146, UK) followed by 100 μg of IV L-T_4 _until they regained their vital functions and were able to take oral medications. The choice for IV or oral route of administration of L-T_4 _was not by design but was based on the availability of IV T_4_, always with an intention to treat with the latter. All patients received 100 mg of IV bolus of hydrocortisone followed by infusion at a rate of 4 mg/hour before the start of L-T_4 _therapy until the results of plasma cortisol were available.

Morbidity and mortality were assessed by GCS and APACHE II and SOFA scores. The SOFA score was calculated at admission and every 2 days until discharge or death. The last-day SOFA score was calculated in all cases. For a single missing value, replacement was calculated from the mean of the sum of the results immediately preceding and following the missing values. During the calculation of a score in a given day, the worst values for each parameter were taken.

### Statistical analysis

The SPSS (Statistical Program for Social Sciences) package, Release 10.01 for PC Windows (SPSS Inc., Chicago, IL, USA), was used for data analysis. In addition to descriptive statistics, the chi-square test was used to assess the association between categorical variables. The *t *test was used to compare the means between the continuous variables. Where the data were skewed, a non-parametric test such as the Mann-Whitney *U *test was used for comparison. The multiple linear regression model was used with death as the dependent variable and a number of other variables such as hospital stay, stoppage of L-T_4_, use of IV T_4_, GCS, and SOFA score, and so on, as independent variables. A *P *value of less than 0.05 was used as the criterion of statistical significance. At an alpha of less than 0.05, the study had a power of 90% in detecting the difference in SOFA score on day 3 between the survivors and those who died. All data are presented as mean ± standard deviation (SD) unless otherwise indicated.

## Results

Twenty-three patients (20 women; 87%) of 59.5 ± 14.8 years of age (range, 30 to 89 years) were included in the present study. The mean lag time between the onset of symptoms and hospitalisation was 10.5 ± 9.7 days (range, 1 to 45 days). Nine (39%) patients were diagnosed with hypothyroidism for the first time (*de novo *group) and 14 (61%) were previously diagnosed (defaulter group). Nineteen (83%) patients had primary hypothyroidism and 4 (17%) had secondary hypothyroidism. Of them, 3 had Sheehan syndrome and 1 had non-functioning pituitary adenoma. Fifteen patients presented in the winter and cold exposure was considered to be a major precipitating factor. Sedative hypnotics and lithium overdose were considered to be a precipitating factor in 3 patients and 1 patient, respectively. Infection was the major accompanying comorbidity in 17 (74%) patients, including bacterial pneumonia in 13, urosepsis in 2, and 1 each with viral gastroenteritis and hepatitis. Twelve patients had a history of diuretic use for their edematous states without correct diagnosis of hypothyroidism. Eight patients had tense ascites at the time of presentation, and 2 patients had myxoedema ileus as a presenting manifestation. Sixteen (69%) patients were deeply comatose and 7 were obtunded at the time of presentation. Myxoedematous skin was present in all patients but was more marked in patients with primary hypothyroidism, and hypothermia was accompanied in all patients. The presenting manifestations and clinical profile are summarised in Table 1.

**Table 1:** Clinical and laboratory findings in 23 patients with myxoedema coma

Subject number	Age, years	Gender	Aetiology of hypothyroidism	Precipitating factors	Associated comorbodities	L-T_4 _route	Outcome
1	46	Female	Secondary	Pneumonia	T2DM, complete heart block	Oral	Died
2	70	Female	Primary	Pneumonia	T2DM, nephropathy	Oral	Died
3	70	Female	Primary	Pneumonia, cold exposure	Old stroke, HTN, DIC	Oral	Died
4	58	Male	Primary	Cold exposure, stoppage of L-T_4_	Old Pott's spine, ascites	Oral	Survived
5	70	Female	Secondary	Cold exposure	T2DM, HTN	Oral	Survived
6	72	Female	Primary	Pneumonia, sedative	T2DM, acute renal failure		Died
7	55	Male	Primary	Urosepsis	OSA, HTN, CLD cortical critical care neuropathy	Oral	Survived
8	60	Female	Primary	Cellulitis, pseudomembranous colitis	DCM, CHF, DIC	Oral	Died
9	59	Female	Secondary	Pneumonia	Anaemia, sepsis, shock	Oral	Died
10	45	Female	Secondary	Pneumonia	Anaemia, pericardial effusion		Died
11	42	Female	Primary	Sedative, cold exposure, hypoglycaemia	Septic shock, respiratory failure	Oral	Survived
12	48	Female	Primary	Cold exposure	DCM, CHF	Oral	Survived
13	89	Female	Primary	Acute, cold exposure, hypoglycaemia	Sepsis, refractory hypotension	Oral	Died
14	50	Female	Primary	Pneumonia, overdose	Atrial fibrillation, right bundle branch block, DIC, T2DM	Oral	Survived
15	30	Female	Primary		Sepsis, DIC, T2DM	Oral	Died
16	52	Female	Primary	Pneumonia	T2DM, extrahepatic portal vein obstruction, sepsis	IV	Died
17	60	Female	Primary	Cold exposure, pneumonia acute gastroenteritis, viral hepatitis	Bronchial asthma, sepsis, DIC	IV	Died
18	85	Male	Primary	Urosepsis, hypoglycaemia, cold exposure	T2DM, HTN, benign prostatic hyperplasia, chronic kidney disease, DIC, refractory seizures	IV	Died
19	83	Female	Primary	Cerebrovascular accident, pneumonia, sedative, cold exposure	Hypotension, bronchial asthma, OSA	IV	Survived
20	60	Female	Primary	Pneumonia, upper gastrointestinal bleed	HTN, CAD, chronic obstetric airway disease, CLD, T2DM, rheumatoid arthritis	IV	Survived
21	50	Female	Primary	Cold exposure	T2DM, HTN, anaemia, old pulmonary tuberculosis, pericardial effusion	Oral	Survived
22	70	Female	Primary	Cold exposure, urosepsis, sedative	T2DM, HTN, CAD, refractory seizures,	Oral	Survived
23	45	Female	Primary	Cold exposure	DCM, pericardial effusion	Oral	Survived

CAD, coronary artery disease; CHF, congestive cardiac failure; CLD, chronic liver disease; DCM, dilated cardiomyopathy; DIC, disseminated intravascular coagulation; HTN, hypertension; IV, intravenous; L-T_4_, L-thyroxine; OSA, obstructive sleep apnoea; T2DM, type 2 diabetes mellitus.

Biochemistry showed mean (± SD) serum sodium of 134.2 ± 10.4 mEq/L (range, 118 to 160 mE q/L). The mean (± SD) serum T_4 _and TSH in patients with primary hypothyroidism were 19.07 ± 14.59 nmol/L and 68.9 ± 41.5 mU/L, respectively. In secondary hypothyroidism, mean (± SD) serum T_4 _and TSH were 23.04 ± 15.36 nmol/L and 3.17 ± 3.47 mU/L (*p *= 0.08 and 0.005), respectively. The mean (± SD) serum T_4 _in the defaulter group was 18.56 ± 12.68 nmol/L as compared with 22.78 ± 11.52 nmol/L in the *de novo *group (*p *= 0.82). The mean (± SD) random serum cortisol was 390.2 ± 82.1 nmol/L, and 7 were glucocorticoid-deficient. Four patients had associated hypoglycaemia at the time of presentation.

Eleven patients had cardiomegaly on chest x-ray either due to dilated cardiomyopathy (3) or pericardial effusion (8), and 17 had an abnormal electrocardiogram (ECG). The various ECG abnormalities included low voltage complex, sinus bradycardia non-specific ST&T changes, left ventricular hypertrophy, atrial arrhythmias, and bundle branch block in decreasing order. Eleven patients had hypoventilation, 8 had CO_2 _narcosis, and 13 had coagulopathy.

Those who stopped L-T_4 _(defaulter group) presented early to the hospital and had advanced manifestations as opposed to those who presented for the first time (*de novo *group) (7.5 ± 5.9 and 15.1 ± 12.8 days, respectively; *p *= 0.06). The clinical manifestations in the defaulter group were more severe as compared with the *de novo *group: bradycardia (heart rate of 60 ± 15 beats per minute versus 82 ± 12 beats per minute; *P *= 0.002), lesser score in GCS (6.0 ± 3.0 versus 8.1 ± 3.5; *P *= 0.18), number of patients requiring mechanical ventilation (71.4% versus 22.2%; *P *= 0.036), and higher mortality (10 versus 2; *p *= 0.036).

There was no significant difference among any parameters between primary and secondary hypothyroidism except that the patients with primary hypothyroidism had more severe skin manifestations, higher TSH level, and relatively lower serum T_4 _level.

Only 11 (45%) out of these 23 patients could survive, with a mean duration of hospital stay of 15.9 ± 18.9 days (range, 2 to 90 days) after the start of treatment. Nine (50%) out of 18 patients who received oral T_4 _died, whereas 3 out of 5 (60%) died in the IV T_4 _group (*p *= 0.782). However, there was no difference in clinical and biochemical parameters in the IV or oral T_4 _group. Although more patients receiving IV T_4 _had sepsis, increased need for mechanical ventilation, longer duration of hospital stay, and higher mortality, none of these could reach statistical significance.

The most common organ dysfunction at presentation was respiratory failure, and the most common new organ dysfunction during hospital stay was coagulopathy. Causes of death included sepsis, upper gastrointestinal bleed, and respiratory failure. The various factors associated with increased mortality were hypotension (*r *= 0.51; *p *= 0.01) and bradycardia (*r *= 0.44; *p *= 0.03) at presentation, need for mechanical ventilation (*r *= 0.65; *p *= 0.00), hypothermia unresponsive to treatment (*r *= 0.51; *p *= 0.01), sepsis (*r *= 0.50; *p *= 0.01), stoppage of L-T_4 _(*r *= 0.48; *p *= 0.01), intake of sedative drugs (*r *= 0.47; *p *= 0.02), lower GCS (*r *= 0.45; *p *= 0.03), higher APACHE II score (*r *= 0.51; *p *= 0.04), and higher SOFA score (*r *= 0.51; *p *= 0.00) (Table 2).

**Table 2:** Factors predicting mortality in survivors and non-survivors

	Survivors (*n *= 11)	Non-survivors (*n *= 12)	*P *value
Age, years	57.36 ± 12.55	61.50 ± 16.97	0.517
Lag time, days	10.64 ± 6.39	10.33 ± 12.29	0.942
Stoppage of L-thyroxine	4	10	0.036^a^
Use of sedatives	7	12	0.037^a^
Heart rate, beats per minute	77.45 ± 14.45	61.25 ± 18.13	0.028^a^
Mean blood pressure, mm Hg	117.64 ± 24.88	90.45 ± 32.28	0.039^a^
Thyroxine, nmol/L	19.84 ± 19.45	23.42 ± 14.46	0.616
Thyroid-stimulating hormone, mU/L	56.99 ± 39.40	57.87 ± 51.99	0.964
Glucocorticoid deficiency	2	5	0.371
Sepsis	6	1	0.027^a^
Unresponsive hypothermia	5	10	0.027^a^
Mechanical ventilation	2	10	0.003^a^
Intravenous thyroxine	2	3	0.9
Glasgow Coma Scale	8.55 ± 3.39	5.50 ± 2.47	0.022^a^
APACHE II score	12.09 ± 4.95	18.08 ± 8.02	0.045^a^
SOFA score, baseline	8.5 ± 3.1	6.6 ± 3.9	0.22
SOFA score, minimum	1.45 ± 1.04	7.58 ± 3.00	0.000^a^
SOFA score, maximum	7.36 ± 3.38	15.17 ± 1.99	0.000^a^
Hospital stay, days	20.73 ± 24.67	11.42 ± 11.09	0.249

APACHE II, Acute Physiology and Chronic Health Evaluation II; SOFA, Sequential Organ Failure Assessment.

**Figure 1:**
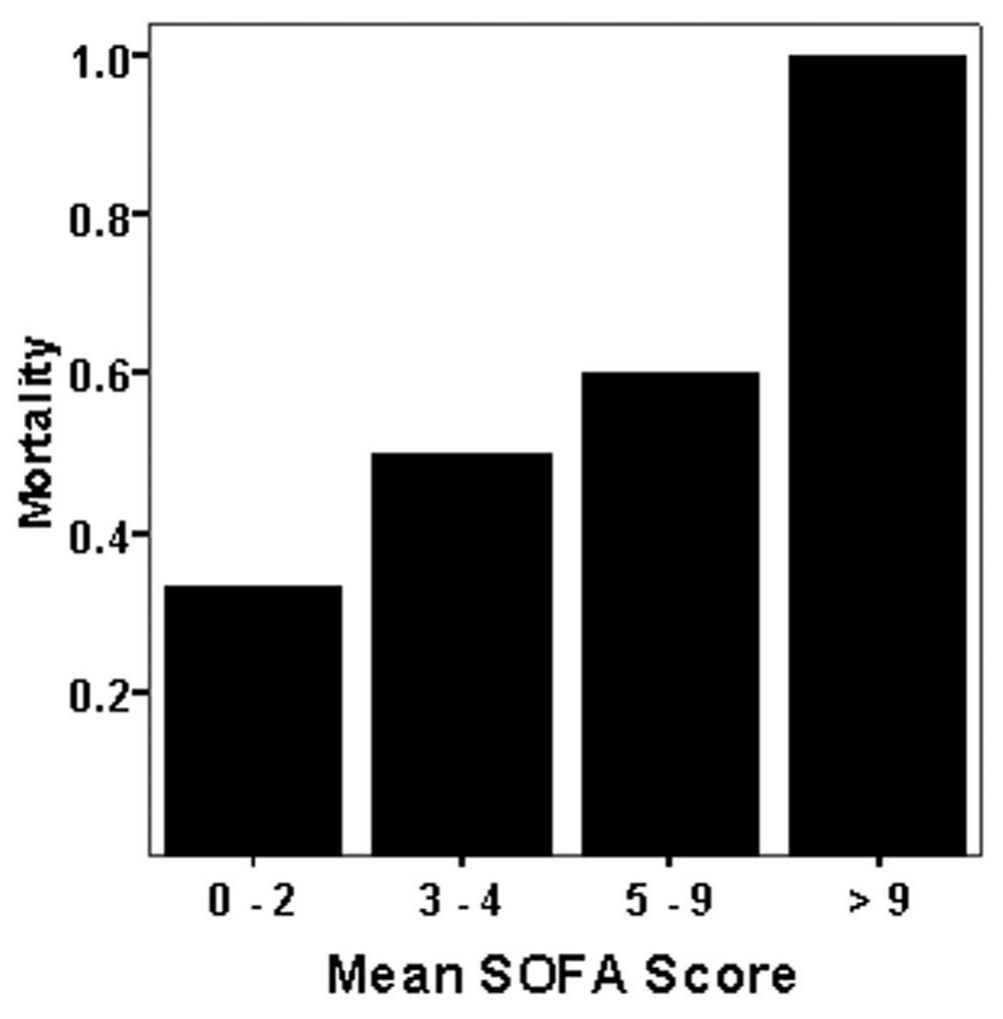
Mean Sequential Organ Failure Assessment (SOFA) score and mortality in myxoedema coma. The higher the SOFA score, the higher the mortality.

**Figure 2:**
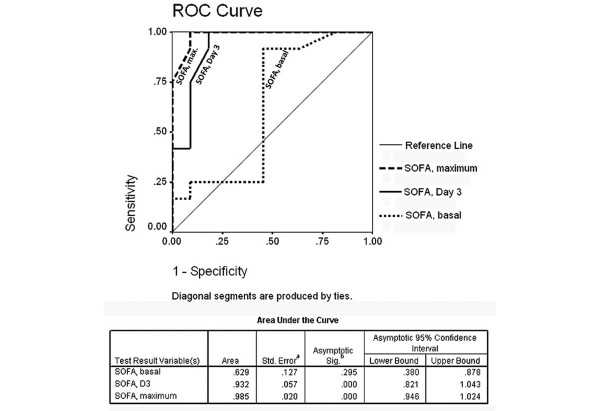
ROC curve showing sensitivity & specificity of various SOFA scores.

On analysing the different prediction models of morbidity and mortality, APACHE II score and GCS had a significant difference between survivors and non-survivors. However, the SOFA prediction module at baseline was not different between the two groups. The baseline and day 3 SOFA scores of greater than or equal to 6 predicted mortality with a sensitivity of 91.7% and a specificity of 100%. Similarly higher the means SOFA score higher was the mortality (Figure 1) All of the prediction modules are summarised in Table 3, and receiver operating characteristic analysis is drawn to assess the area under the curve for the SOFA scores (Figure 2).

**Table 3:** Sensitivity and specificity of SOFA score, GCS, and APACHE II score in predicting mortality in myxoedema coma patients

	Score	Died	Survived	Sensitivity (percentage)	Specificity (percentage)	Positive PV (percentage)	Negative PV (percentage)	Accuracy (percentage)
SOFA score, baseline	≥6	11/12	5/11	91.7	54.5	68.8	85.7	73.9
SOFA score, day 3	≥6	12/12	2/11	100	81.8	85.7	100	91.3
SOFA score, maximum	≥12	12/12	1/11	100	90.9	92.3	100	95.7
SOFA score, delta	≥3.5	9/12	0/11	75.0	100	100	78.6	87.0
SOFA score, mean	≥7	12/12	0/11	100	100	100	100	100
GCS	≤8	11/12	4/11	91.7	63.6	73.3	87.5	78.3
APACHE II score	≥15	8/11	3/11	66.7	72.7	72.7	66.7	69.6

APACHE II, Acute Physiology and Chronic Health Evaluation II; GCS, Glasgow Coma Scale; PV, predictive value; SOFA, Sequential Organ Failure Assessment.

## Discussion

The present study showed that patients who were previously diagnosed and non-compliant with the L-T_4 _treatment had more severe manifestations at presentation and higher mortality in comparison with those who were diagnosed as having hypothyroidism for the first time. The aetiology of hypothyroidism (primary versus secondary) and route of administration of L-T_4 _(oral versus IV) had no influence on outcome. Among various outcome prediction models for critical care illness, the SOFA score was found to correlate best with mortality in these patients.

As hypothyroidism is more common in elderly women, most of our patients were older females (87%) [4]. As in previous reports, the majority of our patients presented in the winter and hypothermia was a frequent accompaniment [4] as cold weather lowers the threshold for encephalopathy in patients with hypothyroidism and this is possibly attributed to the failure of thermoregulatory compensatory mechanisms. In agreement with the published literature, chest and genitourinary infections were the most common comorbidities and/or precipitating factors in the present study [1,3,5,6]. Nearly half of our patients were inappropriately treated with diuretics for edematous state by primary care physicians and that masked the myxoedematous manifestations and posed a difficulty in making an early diagnosis of hypothyroidism.

The prevalence of secondary hypothyroidism in myxoedema coma has been reported to be 5% to 25% [5,7,8]. In our study, 4 (18%) patients had secondary hypothyroidism and all had hypothyroid encephalopathy as a presenting manifestation of their pituitary disease. Due to the paucity of cases, none of the previous studies except one had compared the clinical parameters in primary and secondary hypothyroid patients with myxoedema coma [5]. As expected, the myxoedematous manifestations were very subtle and these subjects had relatively higher T_4 _levels as compared with patients with primary hypothyroidism as TSH-independent production of T_4 _continues in patients with secondary hypothyroidism [10].

An appreciable difference in presenting manifestations, laboratory parameters, and outcome was observed in those who were defaulters as opposed to those who presented *de novo *as having myxoedema coma. However, this issue has not been examined in earlier studies. The defaulter patients had a lower mean heart rate and relatively lower serum T_4 _and the majority of them required mechanical ventilation and had a higher mortality. They also had lower scores on GCS, comparable APACHE II scores, and showed better SOFA scores in comparison with the patients who were diagnosed as hypothyroid at the first presentation of myxoedema coma. This may be attributed to the fact that the non-compliant patients had absolute deficiency of thyroid hormones as compared with those presenting for the first time as having myxoedema coma but who possibly had borderline thyroid hormone reserve.

The dose and route of administration of L-T_4 _in myxoedema coma have always been a matter of debate. The available literature on thyroid hormone replacement therapy in patients with myxoedema coma comprises three categories: IV T_4_, oral T_4_, and oral or IV T_3 _[11]. IV administration of T_4 _has certain advantages, including predictable effect, early saturation of binding sites, and swift replenishment of the thyroid hormone pool [11]. In spite of the above-mentioned advantages with IV T_4_, it has been refuted by other investigators that oral administration of L-T_4 _is associated with variable but quick clinical response, even in patients with myxoedema ileus [11-13]. The recommended initial IV T_4 _bolus dose varies from 100 to 500 μg followed by daily maintenance of 50 to 100 μg until sensorium improves [5,14-16]. However, the cause of coma in all patients may not be uniform, and minor precipitating illness or comorbidity can lead to coma. Therefore, not every patient will require a large loading dose [6,17,18]. Similarly, in last four decades, there has been a considerable change in supportive medical care, thereby decreasing the role of high-dose T_4_. Given the total body store, daily T_4 _production rate, the associated cardiac comorbidities, and the fact that the usual IV loading dose of T_4 _was approximately one half to two thirds of the oral dose, we were compelled to use 200 μg of L-T_4 _as the IV loading dose. We preferred an oral dose of 500 μg as the loading dose since a dose of more than 500 μg/day of oral L-T_4 _was associated with a fatal outcome within 1 month of treatment in one study [6]. This type of approach is supported by a recent publication by Wartofsky [4]. None of the available studies had compared IV T_4 _with oral T_4_. Due to the low availability of IV T_4 _in our country, the present study provides an opportunity not by design, but by default, to analyse this issue. However, only five patients could receive IV T_4 _and this is a small number to make any definitive conclusion. Coincidentally, the patients receiving IV T_4 _were more critically ill than the oral T_4 _group, but mortality could not reach statistical significance between the two groups.

The proponents of T_3 _therapy argue that the onset of action of T_3 _is rapid, that peripheral conversion of T_4 _to T_3 _is hampered in the sick hypothyroid state, and that it has an earlier beneficial effect on neuropsychiatric symptoms as it crosses the blood-brain barrier more rapidly than T_4 _does [4]. However, due to the non-availability of T_3_, we had not used it in our patients.

Patients with long-standing hypothyroidism may have associated glucocorticoid deficiency; therefore, glucocorticoid support is recommended [11]. In our study, all patients received glucocorticoid therapy, but there was no difference in outcome between glucocorticoid-deficient and glucocorticoid-sufficient subjects.

Prediction of outcome is important both in emergency services and intensive care units. Currently available outcome prediction models such as APACHE II score, GCS, SAPS (Simplified Acute Physiology Score), and MPM (Mortality Probability Model) systems calculate a prediction based on values taken in first 24 hours of admission, but not later [19,20]. They are best suited for a mixed pool of patients suffering from a variety of disease conditions, but their validity on a day-to-day basis or for a homogenous group of patients such as those with myxoedema coma is questionable. Unlike these models, the SOFA score is validated for assessing and monitoring organ dysfunction because organ failure is a continuous process rather than an 'all or none' phenomenon [20,21]. The initial SOFA score can be used to predict the degree of organ dysfunction or failure present at admission, delta SOFA score during hospital stay, and total maximum SOFA score represents the cumulative organ dysfunction experienced by the patients [20-22]. In our experience, this was the best module for predicting mortality and morbidity.

The prognosis of patients with myxoedema coma is difficult to predict due to the rarity of the condition. Before 1964, the mortality rate was as high as 80% [23]. The mortality rate in the present study was 52.2%, which is similar to that reported by Arlot and colleagues [13]. The improvement in outcome is attributed to early diagnosis, better supportive care, and use of IV T_4_. In previous studies, poor predictors of outcome included advanced age, bradycardia, persistent hypothermia, level of sensorium, and high APACHE II score at presentation [5,11,22,24]. Our study is in agreement with previous studies in this aspect, except advanced age, which was comparable between survivors and non-survivors. Additionally, low mean blood pressure, requirement for mechanical ventilation, precipitation of myxoedema coma by use of sedatives, accompanying sepsis, and baseline and mean SOFA scores of greater than or equal to 6 were predictive of mortality.

## Conclusion

The patients who were non-compliant to L-T_4 _therapy had earlier presentation, more severe manifestations, and higher mortality compared with *de novo *subjects. Outcome was not influenced by aetiology or route of administration of L-T_4_, and SOFA score was the best outcome-predicting model.

## Key messages

• L-Thyroxine treatment defaulters had more florid manifestations and poorer outcomes as compared with those who were diagnosed for the first time.

• Sepsis is a major accompanying comorbidity in patients with myxoedema coma and usually culminates in death.

• Outcome was not influenced by the aetiology (primary versus secondary) or the route of administration of L-thyroxine (intravenous versus oral).

• Of the available predictor modules, Sequential Organ Failure Assessment was more effective than the others.

## Abbreviations

APACHE II = Acute Physiology and Chronic Health Evaluation II; ECG = electrocardiogram; GCS = Glasgow Coma Scale; IV = intravenous; SD = standard deviation; SOFA = Sequential Organ Failure Assessment; T_3 _= triiodothyronine; T_4 _= thyroxine; TSH = thyroid-stimulating hormone.

## Competing interests

The authors declare that they have no competing interests.

## Authors' contributions

PD contributed to patient management, data collection, and analysis and wrote the manuscript. AB conceived the idea of the study, designed the study, contributed to patient management and data collection, and wrote and edited the manuscript. SRM contributed to statistical analysis, patient management, and data collection. SB contributed to patient management and data collection. NS contributed to patient management. RR contributed to patient management and manuscript editing. All authors read and approved the final manuscript.

## References

[B1] Wall CR (2000). Myxoedema coma: diagnosis and treatment. Am Fam Physi.

[B2] Santiago R, Rashkin MC (1990). Lithium toxicity and myxoedema coma in an elderly woman. J Emerg Med.

[B3] Galofré JC, García-Mayor RV (1997). Densidad de incidencia del coma mixedematoso. Endocrinologia.

[B4] Wartofsky L (2006). Myxoedema coma. Endocrinol Metab Clin N Am.

[B5] Rodríguez I, Fluiters E, Pérez-Méndez LF, Luna R, Páramo C, García-Mayor RV (2004). Factors associated with mortality with myxoedema coma: prospective study in 11 cases treated in a single institution. J Endocrinol.

[B6] Yammamoto T, Fukuyama J, Fujoysh A (1999). Factors associated with mortality of myxoedema coma. Thyroid.

[B7] Cullen MJ, Mayne PD, Sliney I (1979). Myxoedema coma. Ir J Med Sci.

[B8] Reinhardt W, Mann K (1997). Incidence, clinical picture and treatment of hypothyroid coma: results of a survey. Med Klin.

[B9] Arafah BM (2006). Hypothalamic pituitary adrenal function during critical illness: limitations of current assessment methods. J Clin Endocrinol Metab.

[B10] Larsen K, Melmed P Central hypothyroidsim. Williams Textbook of Endocrinology.

[B11] Jordan RM (1995). Myxoedema coma: pathophysiology, therapy and factors affecting prognosis. Med Clin North Am.

[B12] Read DG, Hays MT, Hershman JM (1970). Absorption of oral thyroxine in hypothyroid and normal man. J Clin Endocrinol Metab.

[B13] Arlot S, Debussche X, Lalau JD, Mesmacque A, Tolani M, Quichaud J, Fournier A (1991). Myxoedema coma: response of thyroid hormones with oral and intravenous high-dose L-thyroxine treatment. Intensive Care Med.

[B14] Holvey DN, Goodner CJ, Nicoloff JT, Dowling JT (1964). Treatment of myxoedema coma with intravenous thyroxine. Arch Intern Med.

[B15] Nicoloff JT, LoPresti JS (1993). Myxoedema coma: a form of decompensated hypothyroidism. Endocrinol Metab Clin North Am.

[B16] Ridgway EC, McCammon JA, Benotti J, Maloof F (1972). Acute metabolic response in myxoedema to large doses of intravenous L-thyroxine. Ann Intern Med.

[B17] Khaleeli AA (1978). Myxoedema coma. A report of five successfully treated cases. Postgrad Med J.

[B18] Pereira VG, Haron ES, Lima-Neto N, Medeiros-Neto GA (1982). Management of myxoedema coma: report of three successfully treated cases with nasogastric or intravenous administration of trioidothyronine. J Endocrinol Invest.

[B19] Knaus WA, Draper EA, Wagner DP, Zimmarman JE (1985). APACHE II: a severity of disease classification system. Critical Care Med.

[B20] Ferreira FL, Bota DP, Bross A, Mélot C, Vincent JL (2001). Serial evaluation of the SOFA score to predict outcome in critically ill patients. JAMA.

[B21] Vincent JL, Moreno R, Takala J, Willatts S, De Mendonça A, Bruining H, Reinhart CK, Suter PM, Thijs LG (1996). The SOFA (Sepsis-related Organ Failure Assessment) score to describe organ dysfunction/failure. On behalf of the Working Group on Sepsis-Related Problems of the European Society of Intensive Care Medicine. Intensive Care Med.

[B22] Moreno R, Vincent JL, Matos R, Mendonça A, Cantraine F, Thijs L, Takala J, Sprung C, Antonelli M, Bruining H (1999). The use of maximum SOFA score to quantify organ dysfunction/failure in intensive care. Results of a prospective, multicentre study. Working Group on Sepsis related Problems of the ESICM. Intensive Care Med.

[B23] Forester CF (1963). Coma in myxoedema. Report of a case and review of world literature. Arch Intern Med.

[B24] Haylander B, Rosenquist U (1985). Treatment of myxoedema coma, factors associated with fatal outcome. Acta Endocrinologica.

